# Reduced IGF-1 Levels Following Clomiphene Treatment for Male Hypogonadism

**DOI:** 10.1210/jendso/bvaf078

**Published:** 2025-05-03

**Authors:** Nikita Mogar, Dongyun Zhang, Anthony P Heaney

**Affiliations:** Department of Medicine, David Geffen School of Medicine, UCLA Medical Center, University of California, Los Angeles, CA 90095, USA; Department of Medicine, David Geffen School of Medicine, UCLA Medical Center, University of California, Los Angeles, CA 90095, USA; Department of Medicine, David Geffen School of Medicine, UCLA Medical Center, University of California, Los Angeles, CA 90095, USA; Department of Neurosurgery, David Geffen School of Medicine, UCLA Medical Center, University of California, Los Angeles, CA 90095, USA

**Keywords:** IGF-1, clomiphene, citrate, testosterone, growth, hormone

## Abstract

**Context:**

The selective estrogen receptor modulator clomiphene stimulates pituitary-derived gonadotropins to generate sex steroids including estrogen. Estrogen activates SOCS-3, which can inhibit growth hormone-directed JAK/STAT signaling to reduce serum insulin-like growth factor (IGF)-1 levels.

**Objective:**

We sought to examine the effects of clomiphene therapy on IGF-1 levels in nonacromegalic male patients treated with clomiphene for underlying hypogonadism.

**Methods:**

We identified 20 male subjects with hypogonadism treated with clomiphene citrate for at least 3 months. These patients were treated in an ambulatory, academic, tertiary medical center. The 20 male patients ranged from 27 to 76 years of age and hypogonadism was due to several etiologies, including prolactinomas, clinically nonfunctioning pituitary tumors, Rathke cleft cysts, colloid cysts, or idiopathic causes. Clomiphene citrate 50 mg 3 days per week was administered for a minimum of 3 months. IGF-1 was measured by liquid chromatography-mass spectroscopy before and after clomiphene therapy.

**Results:**

Fifteen of 20 (75%) of hypogonadal men treated with clomiphene exhibited a decrease in median (IQR) serum IGF-1 levels of −0.60 (−1.2-0.0) (*P* < .01). Two of the 20 patients (10%) exhibited a decrease in IGF-1 >2 SD below their age- and sex-matched mean value.

**Conclusion:**

Clomiphene therapy can result in a significant reduction in serum IGF-1 levels in some treated hypogonadal men. Given that the decrease in IGF-1 can be >2 SD in some patients and potentially clinically significant, we recommend interval monitoring of serum IGF-1 levels and symptoms of growth hormone deficiency in patients with hypogonadism treated with clomiphene citrate.

Clomiphene is a selective estrogen receptor modulator that stimulates production of the pituitary gonadotropins, follicle-stimulating hormone (FSH) and luteinizing hormone (LH) [1]. In light of its action to stimulate estrogen and progesterone, it is approved to induce ovulation in women with polycystic ovarian syndrome where success rates approach 50% [2]. It is also used off-label to increase testosterone production in male patients with hypogonadism and has the advantage that it does not cause testicular atrophy [3].

Prior studies have demonstrated that estrogen negatively regulates growth hormone (GH) signaling by activating SOCS-3 to inhibit JAK/STAT signaling and has been shown to reduce insulin-like growth factor (IGF)-1 levels by 40% to 80% in healthy adult men with intact pituitary function [4, 5]. Further studies have confirmed similar effects in postmenopausal women treated with large doses of oral estrogen resulting in decreased IGF-1 levels [6]. Additionally, estrogen stimulates the synthesis of the GH and IGF binding proteins, GHBP and IGFBP, which alters GH action to lower IGF-1 levels [4].

As further evidence of its action to lower IGF-1, clomiphene has been shown to normalize IGF-1 levels in 7/16 (44%) patients with acromegaly and was associated with a concomitant 209% increase in total testosterone (TT) levels [7]. However, literature on the potential actions of clomiphene to alter IGF-1 levels in normal males treated for hypogonadism is quite sparse.

This retrospective study sought to assess the effect of clomiphene treatment on IGF-1 levels in a consecutive series of male patients treated with clomiphene for central hypogonadism.

## Materials and Methods

### Study Design, Setting, and Population

Under institutional review board approval, using encryption password protection software and a secure network server to store data, we interrogated the XDR database, a research tool that contains patient data from all University of California (UC) medical campuses. We identified 99 male UCLA patients aged 18 and older who had been prescribed clomiphene citrate 50 mg by a single investigator (A.P.H.) for a minimum of 3 months between 2012 and 2022.

**Figure 1.**
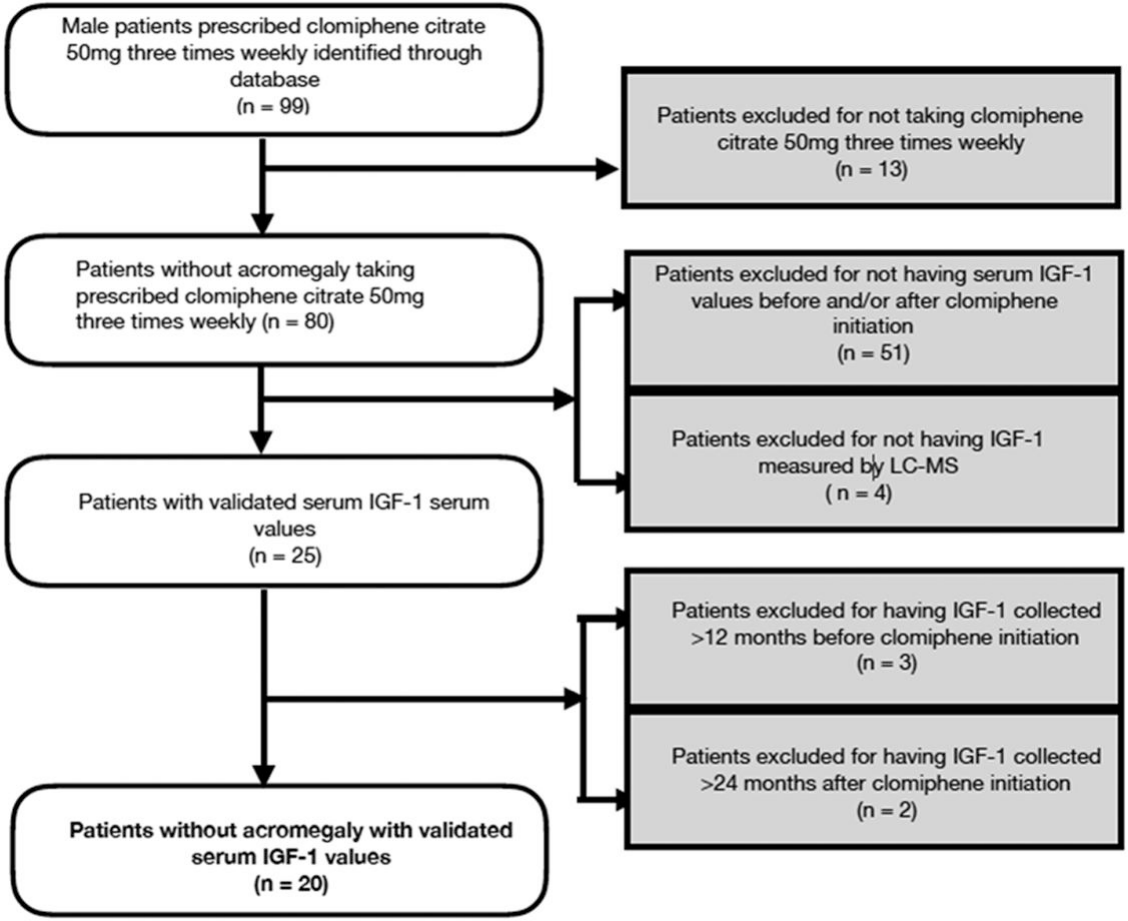
Key inclusion and exclusion criteria.

Seventy-nine of 99 patients were excluded from the study: 13 because they did not take the clomiphene as prescribed, 51 did not have documented IGF-1 levels prior to and after taking clomiphene, 4 had IGF-1 measured by a method other than liquid chromatography-mass spectroscopy (LC-MS), 6 patients who had a diagnosis of acromegaly, and 5 patients who did not have IGF-1 levels collected within 12 months before and within 24 months after clomiphene treatment (Fig. 1).

The remaining 20 male subjects included in the study ranged from 27 to 76 years of age (Table 1). They had all taken clomiphene citrate 50 mg 3 days per week for at least 3 months and had documented IGF levels measured by LC-MS on at least 1 occasion within 12 months before and within 24 months after clomiphene treatment. Fifteen of the 20 study patients (75%) had had space-occupying lesions of the pituitary, 5 had prolactinomas (all of whom had normal serum prolactin levels on cabergoline therapy), 6 had clinically nonfunctioning pituitary tumors, 5 had idiopathic hypogonadism (IH) with no imaging abnormalities, 2 had silent corticotroph tumors, 1 had a Rathke cleft cyst, and 1 had a colloid cyst. Four patients (#5, #9, #18, and #19) were on replacement therapy, 4 were receiving a stable dose of thyroid replacement for at least 6 months with normal free thyroxine levels, and 1 of these 4 patients was also receiving glucocorticoid replacement (#9). Four out of 5 patients with prolactinomas were being treated with cabergoline and had been on a stable dose for at least 6 months (Table 2)

**Table 1.** Clinical characteristics of 20 male patients treated with clomiphene from 2012 to 2022

Median age—years (IQR)	47 (38.5-55)
Median duration of clomiphene treatment—months (IQR)	14.5 (− 10-25)
Patients on pituitary replacement therapy or other pituitary therapy (%)	6 (30%)
Levothyroxine (%)	4 (20%)
Hydrocortisone (%)	1 (5%)
Cabergoline (%)	5 (25%)
Median baseline IGF-1 SD (IQR)	0.10 (−0.5-0.85)
Median IGF-1 SD (IQR) after the initiation of clomiphene	−0.55 (−1.2-0.2)
Median change in IGF-1 SD (IQR) after the initiation of clomiphene	−0.60 (−1.2-0.0)
Median baseline total testosterone (ng/dL) (IQR)	251.2 (193.1-375.5)
Median total testosterone (ng/dL) (IQR) after initiation of clomiphene	574.8 (366.5-755.5)
Median change in total testosterone (ng/dL) (IQR)	219 (100.5-363.7)
Median baseline free testosterone (pg/mL) (IQR)	57.5 (35.6-87.3)
Median free testosterone (pg/mL) (IQR) after initiation of clomiphene	98 (64.4-136.1)
Median change in free testosterone (pg/mL) (IQR)	23.2 (−4.9-91.4)

Abbreviations: ID, insufficient data; IQR, interquartile range.

**Table 2.** Clinical characteristics of 20 patients treated with clomiphene citrate from 2012 to 2022

	Etiology of Secondary Hypogonadism (baseline tumor size [cm], TNTS)	PRT, CBG	Clomiphene dose (months)	Pre-CC IGF-1 SD (months)	Post-CC IGF-1 SD (months)	Pre-CC TT (months) SHBG (months)	Post-CC TT (months)	Pre-CC FT (months	Post-CC FT (months)	Baseline FSH, LH	Pre-CC E2 (months)	Post-CC E2 (months)	Sxs	Improved?
Patient 1	Colloid Cyst (4.5, N [EVD])	—	50 mg TIW (24)	0.8 (11)	−0.4 (3)	535 (7) 37 (7)	855.6 (3) N/A	95 (6)	136.1 (3)	4.6, 5.0	N/A	59 (3)	Low libido	N
Patient 2	CNFT (0.3, N)	—	50 mg TIW (21)	0.1 (0.3)	0.2 (6) 0.0 (9)	375 (0.2) N/A	582.5 (6) N/A	N/A	157.5 (6)	5.8, 5.2	16 (0.2)	38 (6)	Fatigue	N
Patient 3	CNFT (1.2, Y)	—	50 mg TIW (26)	1.2 (1)	−1.2 (5)	239.2 (0.5) N/A	646 (22) 21 (22)	57.9 (1)	155.5 (10)	NA, 4	N/A	N/A	Fatigue	Y
Patient 4	CNFT (0.5 N)	—	50 mg TIW (10)	0.2 (2)	−1.3 (2) −1.4 (5) −1.3 (9)	445 (18) 33 (18)	655 (5) N/A	92 (18)	77.6 (5)	NA, 2.8	35 (18)	19 (2)	Fatigue, low libido	N
Patient 5	Silent corticotroph adenoma (2.5, Y)	T	50 mg TIW (13)	−0.74 (1)	−0.047 (10)	267 (1) 27.6 (5)	525 (10) N/A	82.5 (1)	185.3 (11)	NA, NA	N/A	N/A	Fatigue, low libido	N
Patient 6	Idiopathic (0.2, N)	—	50 mg TIW (14)	0.3 (1)	0.7 (13)	512 (12) N/A	725 (2) 25 (2)	141 (12)	151 (7)	3.4, 2.3	N/A	N/A	Fatigue	N
Patient 7	CNFT (3.5, Y)	—	50 mg TIW (53)	−0.2 (10)	−0.5 (3)	376 (9) N/A	880 (31) N/A	58.6 (9)	108 (31)	3.1, 2.3	N/A	N/A	Fatigue, low libido	Y
Patient 8	Silent corticotroph adenoma (3.5, Y)	—	50 mg TIW (17)	1.6 (8)	1.5 (10) 0.8 (20)	253.4 (2) N/A	398.2 (10) N/A	29 (2)	36.3 (17)	2.7, 1.7	N/A	N/A	Fatigue	Y
Patient 9	Idiopathic (N)	T/C	50 mg TIW (4)	1.7 (1)	1.1 (4)	178 (0.5) N/A	234 (5) N/A	48.2 (0.5)	43.3 (3)	5.9, 3.2	N/A	N/A	Fatigue, low libido	Y
Patient 10	Idiopathic (N)	—	50 mg TIW (12)	0.9 (0.5)	−0.5 (3) −1.2 (9)	228 (0) N/A	641 (9) 22 (12)	43.2 (0)	134.6 (9)	1.7, 1.3	N/A	29 (12)	Low libido	Y
Patient 11	Idiopathic (N)	—	50 mg TIW (60)	−0.8 (1)	−1.0 (23)	248 (3) 8.4 (3)	567 (5) 13 (6)	N/A	64.4 (5)	0.6, 1.2	N/A	N/A	Fatigue, hair loss	Y
Patient 12	PRLoma (3, N)	CBG 1.5 mg BIW	50 mg TIW (10)	0.1 (3)	0.1 (10)	55 (6) 12 (6)	287 (10) N/A	13.2 (6)	N/A	N/A, N/A	N/A	N/A	Fatigue	N/A
Patient 13	RCC (0.7, Y)	—	50 mg TIW (84)	1.7 (5)	0.5 (11)	725 (0.5) N/A	786 (66) N/A	99.6 (0.1)	66 (11)	1.8, 2.6	N/A	27 (66)	Fatigue, low libido	Y
Patient 14	CNFT (2.7, Y)	—	50 mg TIW (8)	0.3 (12)	−2.3 (9)	309 (8) 20 (8)	344 (9) N/A	42.2 (7)	42.2 (9)	8.7, 3.8	N/A	N/A	Fatigue, low libido	N
Patient 15	Idiopathic (N)	—	50 mg TIW (9)	−0.09 (0.5)	−1.4 (0)	249 (0) N/A	389 (9) 23 (9)	N/A	69.6 (9)	5.6, 6	N/A	N/A	Fatigue	Y
Patient 16	PRLoma (0.8, N)	CBG 1 mg QW	50 mg TIW (13)	0.4 (10)	−0.9 (3) −0.8 (12)	266 (10) 15 (10)	289 (11) N/A	57 (10)	35.3 (11)	N/A, N/A	48 (11)	N/A	Fatigue, low libido	Y
Patient 17	PRLoma (4.5, N)	CBG 2 mg BIW	50 mg TIW (28)	0.4 (3)	0.2 (13)	144 (6) N/A	973 (13) N/A	27 (6)	124 (13)	N/A, N/A	N/A	N/A	Fatigue, low libido	Y
Patient 18	PRLoma (2.7, N)	CBG 1 mg BIWT	50 mg TIW (21)	−0.7 (0.3)	−0.6 (9) −0.9 (20)	178 (0.3) N/A	928 (9) N/A	N/A	88 (20)	N/A, N/A	N/A	N/A	Fatigue	N/A
Patient 19	CNFT (3.2, Y)	T	50 mg TIW (6)	−0.9 (7)	−2.1 (5)	148 (2) 29 (2)	157 (6) 24 (6)	27 (2)	N/A	3.6, 2.4	N/A	N/A	Fatigue	N/A
Patient 20	PRLoma (1.7, N)	CBG 0.5 mg BIW	50 mg TIW (15)	0.9 (4)	1.3 (16)	208.2 (1) N/A	490.1 (3) N/A	71.8 (1)	108.2 (3)	N/A, N/A	N/A	N/A	Low libido	N/A

Reference ranges: SHBG (ref. 13-90 nmol/L); TT (ref. 300-1080 ng/dL); free testosterone (ref. 47-244 pg/mL); estradiol (ref. <39 pg/mL); FSH (ref. 1.6-9.0 mIU/mL); LH (ref. 2.0-12.0 mIU/mL).

Abbreviations: BIW, 2 times weekly; C, glucocorticoid replacement; CC, clomiphene citrate; CBG, cabergoline; CNFT, clinically nonfunctional tumor; EVD, external ventricular drain; FT, free testosterone; N, no; N/A, not applicable; OW, once weekly; PRLoma, prolactinoma; PRT, pituitary replacement therapy; RCC, Rathke cleft cyst; SHBG, sex hormone–binding globulin; Sxs, symptoms; T, thyroid hormone replacement; TIW, 3 times weekly; TT, total testosterone; Y, yes.

### Data and Statistical Analysis

The diagnosis of hypogonadism was confirmed by measurement of early morning (8-9 Am) TT levels by LC-MS for all subjects on at least 2 occasions <300 ng/dL and/or free testosterone <50 pg/mL [8]. The normal range for morning total and free testosterone were (250-1100 ng/dL) and (47-244 pg/mL), respectively. All patients reported fatigue and reduced libido. IGF-1 was measured by LC-MS at Quest laboratories in all subjects, and IGF-1 levels are expressed as SD according to the individual's age- and sex-matched reference ranges.

Patient demographic, clinical, and biochemical data are depicted in Table 2. IGF-1, total, and free testosterone levels before and after clomiphene therapy were compared by the nonparametric t-test, and changes in IGF-1 SD were correlated with total and free testosterone levels using GraphPad Prism software.

## Results

**Figure 2.**
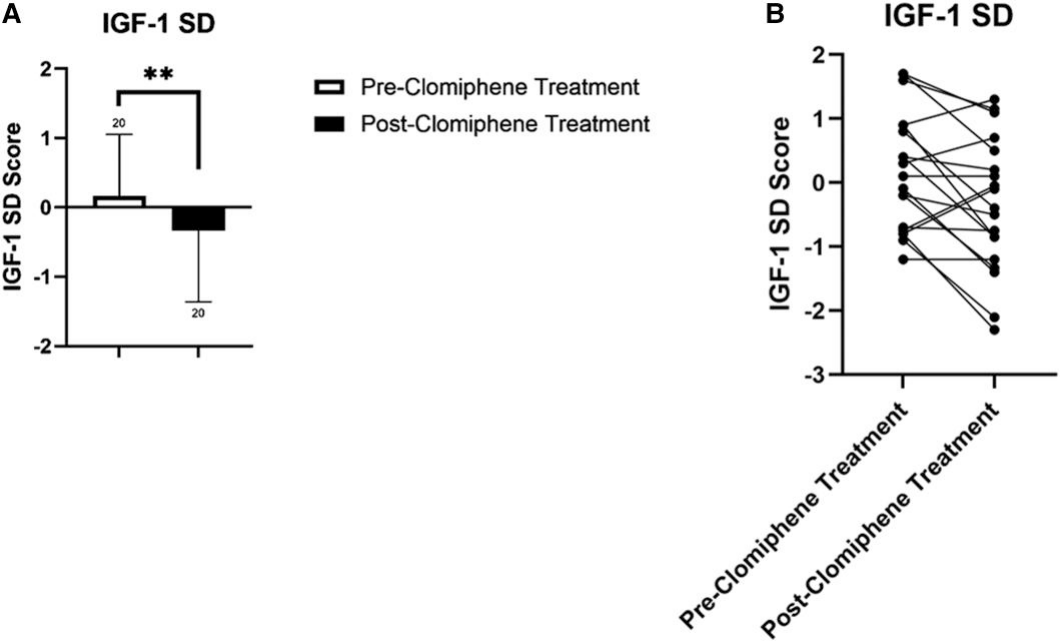
(A) Box plot depiction of trend in IGF-1-SD after clomiphene citrate initiation. **P < .01. (B) Estimation plot depiction of trend in IGF-1-SD before and after clomiphene citrate initiation.

Fifteen of 20 (75%) patients exhibited a decrease in serum IGF-1 levels that ranged from −2.1 SD to −0.1 SD with median change and interquartile range (IQR) in IGF-1 SD of −0.60 (−1.2-0.0) respectively (*P* < .01) (Table 1 and Fig. 2A and 2B). Two of these patients (10% of the total cohort, and 13% of the patients with declines in IGF-1) exhibited a decreased IGF-1 ≥2 SDs below their age- and sex-matched mean value. Three patients (15%) exhibited an increase in IGF-1 SD of +0.693, 0.4, and 0.4-SD, respectively and in 2 patients (10%), IGF-1 levels were essentially unchanged.

**Figure 3.**
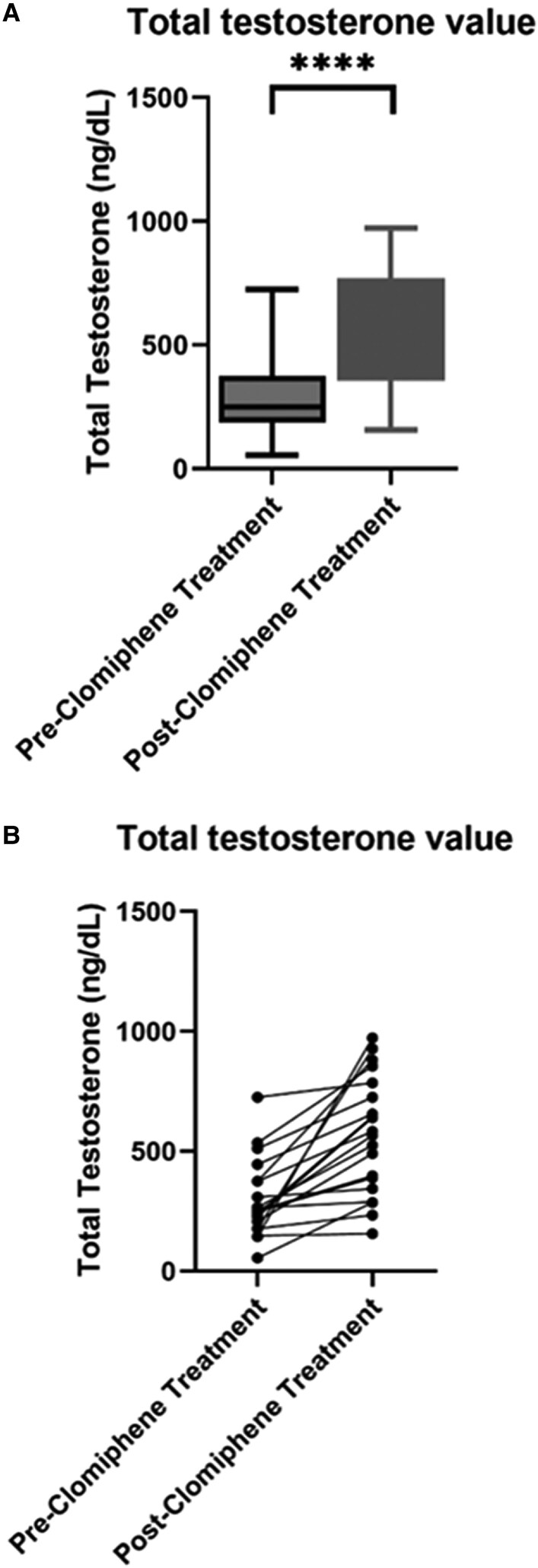
(A) Box plot depiction of trend in total testosterone (TT) after initiation of clomiphene citrate. Pre-TT: total testosterone before clomiphene initiation; Post-TT: total testosterone after clomiphene initiation. (B) Estimation plot depiction of trend in total testosterone (TT) after initiation of clomiphene citrate. Pre-TT: total testosterone before clomiphene initiation; Post-TT: total testosterone after clomiphene initiation. ****P < .001.

All 20 (100%) patients exhibited an increase in their serum TT levels ranging from 9 to 829 ng/dL with a median increase in TT of 216 and IQR (100.5-363.7) (*P* < .0001) (Fig. 3A and 3B). The observed reduction in IGF-1 SD correlated weakly with the fold increase in TT with a correlation coefficient 0.36, though this was not statistically significant (*P* = .12).

**Figure 4.**
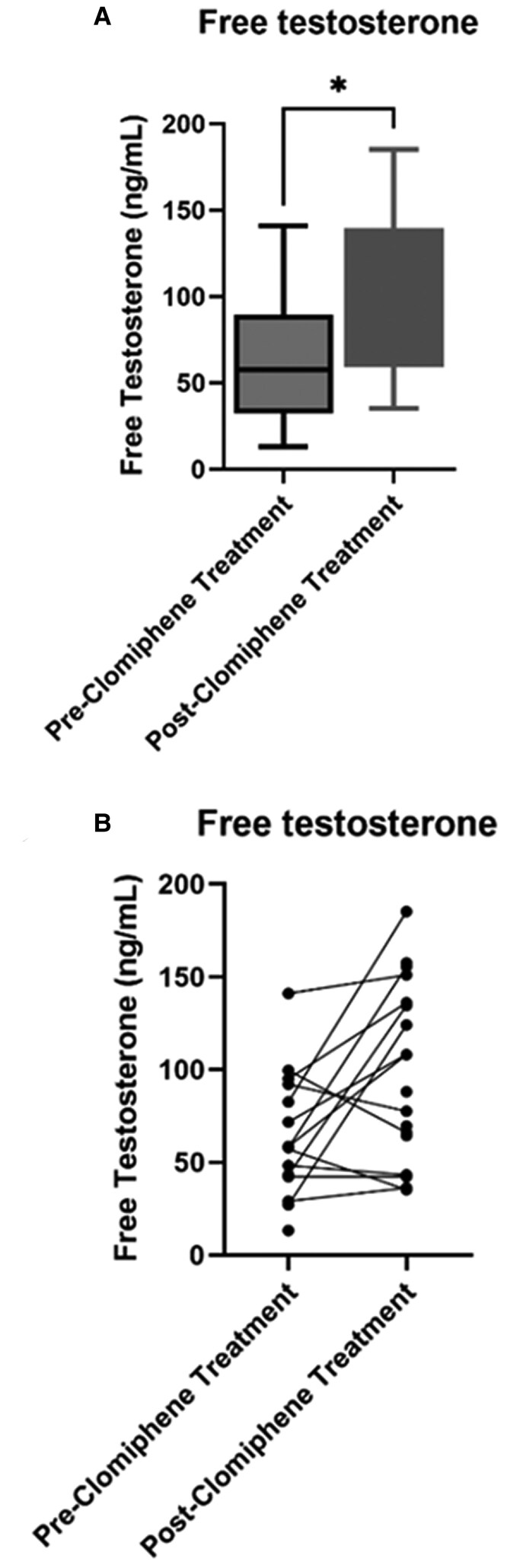
(A) Box plot depiction of trend in free testosterone (FT) after initiation of clomiphene citrate. Pre-FT: free testosterone before clomiphene initiation; Post-FT: free testosterone after clomiphene initiation. (B) Estimation plot depiction of trend in free testosterone (FT) after initiation of clomiphene citrate. Pre-FT: free testosterone before clomiphene initiation; Post-FT: free testosterone after clomiphene initiation. *P < .05.

Nine of these 20 patients (45%) also exhibited an increase in serum free testosterone levels ranging from 10 to 102.8 pg/mL with a median increase of 23.2 and IQR (−4.9-91.4) (*P* < .05) (Fig. 4A and 4B). Four patients (20%) exhibited a decrease in free testosterone levels of 14.4, 4.9, 33.6, and 21.7, respectively, 1 patient had no change in free testosterone, while insufficient samples were available in the remaining 6 patients. The decrease in IGF-1 levels correlated weakly with the fold increase in free testosterone with a correlation coefficient of 0.20, but this was not statistically significant (*P* = .45).

## Discussion

Male hypogonadism is a commonly encountered problem, estimated to affect 2% to 13% of the world's population [9, 10] and causes a wide variety of symptoms including fatigue, poor libido, erectile dysfunction, depressed mood, and decreased muscle mass. Replacement testosterone therapy is an option in symptomatic patients with low testosterone levels, typically defined as an early-morning (8-9 Am) TT <300 ng/dL and/or free testosterone <50 pg/mL [8]. However, side effects can include erythrocytosis, possible hypercoagulability, and hyperlipidemia. Additionally, parenteral testosterone may be challenging for some patients, and skin reactions and/or androgen transfer to partners can occur with topical testosterone preparations, leading to reduced long-term compliance.

Off-label clomiphene use can be an option for some patients where gonadotroph function is relatively preserved, and it is generally well tolerated. Unlike exogenous testosterone therapy, clomiphene preserves male fertility and does not cause testicular atrophy. As discussed in the introduction, clomiphene acts at the hypothalamic level to prevent estradiol-mediated negative inhibition to increase GnRH pulsatility, thereby increasing FSH and LH production [9]. In a study of clomiphene 25 mg used in hypogonadal men for 12 weeks, the testosterone to estradiol ratio increased by 61% [11]. A further meta-analysis of 19 studies including 4 randomized clinical trials, found that total and free testosterone, LH, FSH, and estradiol all increased after clomiphene citrate therapy with improvement in hypogonadal symptoms as measured through the Androgen Deficiency in Aging Males questionnaire [12].

Clomiphene has also been shown to increase sperm concentration [13] although a multicenter randomized controlled trial of 190 couples did not report increased pregnancy rates with clomiphene therapy vs placebo [14, 15].

Studies evaluating long-term clomiphene use in men are reassuring. In a retrospective study of 400 patients treated with clomiphene citrate for a mean ± SD of 25.5 ± 20.48 months, 88% of patients treated for more than 3 years achieved normal TT levels (>300 ng/dL), with 77% experiencing improved symptoms, and only 5/400 (8%) reporting side effects such as mood changes, blurred vision, and breast tenderness with no significant adverse events [16].

This is the first retrospective study examining IGF-1 measurements before and after clomiphene treatment in hypogonadal males. We demonstrated a statistically significant decline in IGF-1 SD levels in the majority of patients after treatment with clomiphene citrate for at least 3 months, and 2 patients exhibited a decline in IGF-1 levels exceeding −2 SD below their age- and sex-matched mean values (patients 14 and 19). In parallel, all of our clomiphene-treated patients exhibited increased TT levels (*P* < .0001). It is important to acknowledge that 15/20 (75%) of our patients had been treated for a pituitary space occupying lesion, though no mass was present at the time of clomiphene therapy. Nonetheless, this is clearly a population of patients that are potentially vulnerable to GH deficiency and may therefore be more susceptible to the GH-blocking actions of clomiphene to lower IGF-1 levels. Interestingly, we did see some variation in the changes in TT and IGF-1 across the various etiologies of hypogonadism. For instance, the greatest increase in TT was seen in the patients with prolactinomas, where 5/5 (100%) exhibited increases in testosterone ranging from 9% to 576% (median 421%). Three of these 5 (60%) had reductions in IGF-1-SD (median % change −28.6% [range −300%-44.4%], median absolute change [IQR] −0.2 [−0.7-0.2]). In contrast the changes seen in 5 patients with IH and 6 patients with clinically nonfunctional tumor (CNFTs) were lower but comparable with median increases in testosterone of (IH: median 56% [range 31-181%] and CNFT: median 51% [range 6-170%]), respectively. Four of 5 (80%) patients with IH had reductions in IGF-1-SD (median % change −323% [range −1455-133%], median absolute change [IQR] −0.6 [−1.7-−0.3]) and 6/6 (100%) patients with CNFTs had reductions in IGF-1 SD (median change −333% [range −750-100%], median absolute change [IQR] −1.35 [−2.4-−0.3]). Of the remaining 4 patients, 2 with silent corticotroph adenomas had increases in testosterone (median 77% [range 57-97%]), and 1/2 (50%) had reductions in IGF-1 SD (median change −21.5% [range −50-93%], median absolute change [IQR] −0.05 [N/A]), 1 patient with a colloid cyst had an increase in TT by 60% and a reduction in IGF-1-SD by 42% (absolute change −1.2-SD), and the final patient with a Rathke cleft cyst had an increase in TT by 8.4% and a reduction in IGF-1-SD by 39% (absolute change −1.2-SD). Although these findings must be interpreted with caution give the small numbers involved, it is interesting that the greatest increase in TT following clomiphene therapy was seen in the patients with prolactinomas, which perhaps infers a greater response given the underlying mechanism for gonadotrope dysfunction is functional rather than destructive as in a space-occupying lesion.

The 2 patients who exhibited the most significant declines in IGF-1 levels following clomiphene therapy had clinically nonfunctioning pituitary tumors, which are often large pituitary lesions at presentation. This raises the possibility that these subjects may have had some pre-existing degree of compromised GH action even though they were maintaining normal age- and sex-matched IGF-1 levels prior to clomiphene therapy. Against that however, other than hypogonadism, pituitary function was intact in 1 of these patients (patient 14), and the other patient (patient 19) was only receiving thyroid replacement therapy with normal free thyroid hormone levels (free thyroxine: 1.0 ng/dL). Furthermore, baseline testosterone levels in patient 14, where IGF-1 levels decreased from −0.8 SD to −2.3 SD, were not exceptionally low at 309 ng/dL, as one typically encounters in pituitary tumor–associated hypogonadism. Similarly in patient 19 who exhibited a decrease in IGF-1 from −0.9 SD to −2.1 SD, TT at baseline was 148 ng/dL and increased to 157 ng/dL after 5 months of clomiphene therapy. Interestingly, symptoms of decreased energy and reduced libido did not change in either of these 2 subjects following clomiphene therapy. The dose and duration of clomiphene did not differ in these 2 patients compared with the rest of the patient population.

Whether the lack of symptomatic improvement in these 2 patients despite increases in TT while on clomiphene was in part related to their significant reductions in IGF-1 levels is unclear, but our studies would support further investigation of this action of clomiphene.

Some studies have shown that testosterone therapy can lead to a dose-dependent increase in serum IGF-1 levels. In 1 study, male patients aged 18 to 35 years old given a range of intramuscular testosterone doses (25-600 mg/week) in combination with gonadotropin-releasing hormone (GnRH) to suppress endogenous testosterone production exhibited a dose-dependent increase in IGF-1 with a 300 and 600 mg testosterone dose [17]. Clearly, this mode of androgen replacement using testosterone itself is different to the use of clomiphene, with its selective estrogen receptor modulator action on GnRH.

Our study was retrospective and our patients did not have formal evaluations of their quality of life and symptoms before and after clomiphene initiation. Therefore, although we have observed statistically significant biochemical changes in IGF-1 levels following clomiphene therapy, we cannot tell if any of these changes are clinically important. Furthermore, symptoms due to reductions in IGF-1 levels could be offset by improvements in androgen levels. Future studies should include a careful assessment of symptoms and quality of life in parallel with serial biochemical measurements of IGF-1 and free and TT measurements before and after clomiphene therapy to clarify this question. Although our study sample size is small given the rarity of these patients, we believe it adds useful knowledge to this field and may spur additional investigation in this area.

In conclusion, we have observed that clomiphene therapy to treat hypogonadism in men can result in a significant reduction in serum IGF-1 levels in some patients, which could contribute to several clinical consequences including fatigue, increased insulin resistance and adipocyte mass, reduced lean muscle and bone mass as well as reduced quality of life. Given the decrease in IGF-1 can be ≥2 SD in some patients and thereby potentially clinically significant, we recommend interval monitoring of serum IGF-1 in patients with hypogonadism treated with clomiphene citrate.

## Data Availability

Original data generated and analyzed during this study are included in this published article or in the data repositories listed in References.

## Abbreviations

CNFT: clinically nonfunctioning pituitary tumor
FSH: follicle-stimulating hormone
GH: growth hormone
GnRH: gonadotropin-releasing hormone
IGF: insulin-like growth factor
IH: idiopathic hypogonadism
LC-MS: liquid chromatography-mass spectroscopy
LH: luteinizing hormone
TT: total testosterone
